# Flow-Cell-Compatible Operando Surface-Enhanced Raman Spectroscopy for Probing Reaction Intermediates during Carbon Dioxide Reduction Reaction

**DOI:** 10.1021/acs.jpclett.6c00902

**Published:** 2026-05-19

**Authors:** Yu-Jhih Shen, Yung-Hsi Hsu, Yu-Chia Chang, Yu-Cheng Liu, Kang-Shun Peng, Chih-Wei Hu, Ying-Rui Lu, Shao-Hui Hsu, Sung-Fu Hung

**Affiliations:** † Department of Applied Chemistry and Center for Emergent Functional Matter Science, 34914 National Yang Ming Chiao Tung University, Hsinchu 300, Taiwan; ‡ Department of Medicinal and Applied Chemistry, Kaohsiung Medical University, Kaohsiung 807, Taiwan; § 57815 National Synchrotron Radiation Research Center, Hsinchu 300, Taiwan; ∥ Taiwan Semiconductor Research Institute, National Applied Research Laboratories, Hsinchu 300, Taiwan

## Abstract

Elucidating reaction intermediates under practically relevant conditions remains a fundamental challenge in electrocatalysis. For an electrochemical CO_2_ reduction reaction (CO_2_RR), reaction mechanisms can differ substantially between conventional H-cells and high-rate flow cells, complicating accurate *operando* characterization. In this study, we develop a flow-cell-compatible operando surface-enhanced Raman spectroscopy platform by integrating silica-coated Au nanorods (AuNRs@SiO_2_) optimized for 785 nm excitation. The plasmonic resonance enhances Raman signals while suppressing fluorescence, and the inert SiO_2_ shell prevents catalytic perturbation, enabling sensitive measurements under practical catalytic conditions. Using a benchmark copper catalyst, we directly detect key transient intermediates, including *CO_2_^–^ and *HOCCOH, that are inaccessible by conventional Raman spectroscopy. Deconvolution of the adsorbed *CO reveals bridge-bonded, low-frequency, and high-frequency components, establishing a quantitative correlation between *CO speciation and C_2_ selectivity. Parallel flow-cell and H-cell comparisons highlight the necessity of realistic mass transport. This work establishes a general *operando* spectroscopic strategy for mechanistic studies under realistic CO_2_RR conditions.

## Graphical abstract

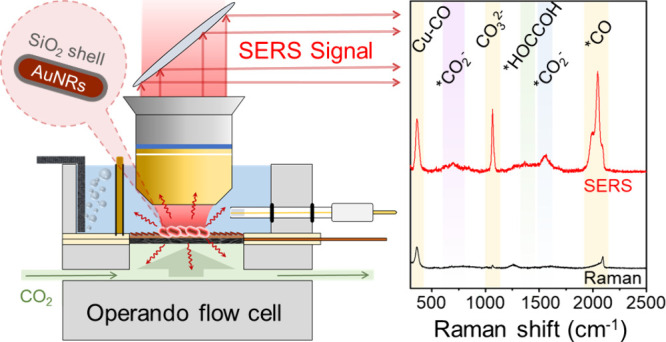

The extensive reliance on fossil fuels has led to substantial CO_2_ emissions, giving rise to critical global challenges such as the greenhouse effect and rising sea levels. Consequently, mitigating atmospheric CO_2_ concentrations has become an urgent priority. Among various emerging technologies,^1–3^ the electrocatalytic CO_2_ reduction reaction (CO_2_RR) has attracted significant attention in recent decades as a promising approach to convert CO_2_ into valuable chemical feedstocks–such as ethylene and ethanol–thereby promoting sustainable carbon cycling.^4–7^ This approach not only enables carbon reutilization and energy storage but also offers a viable pathway to reduce reliance on fossil fuels.^8,9^

To address the inherently low solubility of CO_2_ in aqueous electrolytes in conventional H-type reactors—which significantly limits CO_2_RR performance—a variety of flow-based reactor configurations have been developed.^10–12^ These systems enable the direct delivery of gaseous CO_2_ to the catalytic surface, thereby substantially enhancing both catalytic selectivity and activity. Given that the catalytic behavior and local reaction environment in flow-based reactors differ remarkably from those in conventional setups, it is imperative to re-evaluate the nature of active sites, reaction mechanisms, and surface intermediates under these conditions. This, in turn, highlights the critical need for advanced characterization techniques tailored to flow-based CO_2_RR systems.^13,14^

Over the past decades, a wide range of operando and in situ techniques have been developed to probe catalytic processes, including scanning transmission electron microscopy,^15^ various X-ray-based methods,^12,16,17^ attenuated total reflectance Fourier transform infrared spectroscopy (ATR-FTIR),^18^ Raman spectroscopy,^19^ and surface-enhanced infrared absorption spectroscopy (SEIRAS).^20^ Among these techniques, operando Raman spectroscopy has emerged as a powerful approach for identifying reaction intermediates during electrocatalytic processes,^21–23^ owing to its noncontact, nondestructive nature and, mostly notably, its relatively low sensitivity to water. These advantages render Raman spectroscopy particularly well-suited for investigating electrochemical reactions across a wide range of reactor configurations and operating environments.^24,25^

To grasp more comprehensive insights into reaction intermediates, further enhancement of Raman signals is essential. Surface-enhanced Raman scattering (SERS) is a powerful technique capable of detecting trace chemical species down to the single-molecule level, making it well-suited for operando investigations during CO_2_RR.^26–28^ However, SERS enhancement typically requires metallic substrates exhibiting surface plasmon resonance, such as gold (Au) and silver (Ag).^29–31^ While effective, these noble metals may introduce additional catalytic activity, thereby potentially perturbing the intrinsic catalytic behavior of the system. To overcome this limitation, a strategy has been developed in which gold nanoparticle are coated with an ultrathin, uniform silica shell. This configuration enables efficient Raman signal enhancement of nearby molecular species while effectively preventing direct catalytic participation of the plasmonic metal core. This approach offers a robust platform for operando monitoring of surface intermediates while preserving the intrinsic properties of the catalytic system.^32–35^

Although operando SERS has been successfully applied to CO_2_RR studies in conventional electrochemical configurations, such as H-cells,^36,37^ and operando Raman analysis in gas-fed flow systems has also been developed,^38–41^ the integration of SERS with a realistic flow-cell configuration remains underexplored. This limitation hinders comprehensive investigation of reaction intermediates under practically relevant operating conditions. Herein, we develop a flow-cell-compatible operando surface-enhanced Raman spectroscopy (SERS) platform using SiO_2_ shell-isolated Au nanorods (AuNRs@SiO_2_) optimized for 785 nm excitation, as shown in Figure 1a. This design effectively suppresses fluorescence background while significantly enhancing the Raman signals, thereby enabling the detection of critical reaction intermediates that are inaccessible in conventional H-cell measurements or in the absence of SERS. The operando flow-cell architecture incorporates an ion exchange membrane to separate the catholyte and anolyte compartments, thereby preventing product crossover that could otherwise compromise electrocatalytic performance. Additionally, a gas chamber positioned beneath the cell ensures efficient CO_2_ diffusion to the catalyst surface. The enhanced Raman signals acquired under operando flow-cell conditions allow a detailed analysis of the distinct roles of high-frequency-band (HFB) and low-frequency-band (LFB) CO species in governing C–C coupling pathways and product selectivity during CO_2_ reduction.

**Figure 1:**
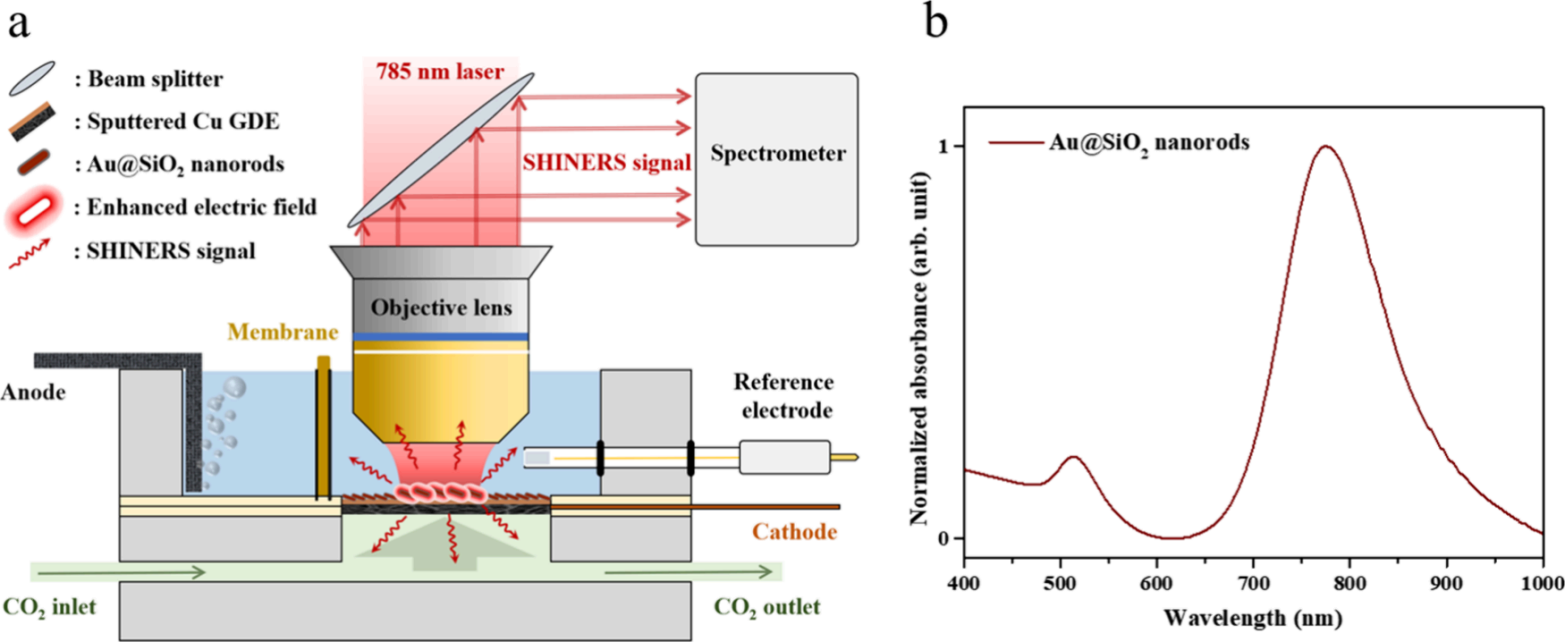
(a) Schematic of the flow-cell-compatible operando surface-enhanced Raman spectroscopy using SiO_2_ shell-isolated Au nanorods. (b) Normalized absorption spectrum of AuNRs@SiO_2_.

To optimize SERS performance under 785 nm excitation, we leveraged the tunable surface plasmon resonance of Au nanorods. The characteristic absorption spectra of the synthesized Au nanorods, spanning from 750 to 870 nm, are shown in Figure S1, and the corresponding synthetic parameters are summarized in Table S1. Au nanorods exhibiting a longitudinal plasmon resonance at 770 nm were subsequently coated with a silica shell to form SiO_2_ shell-isolated Au nanorods (AuNRs@SiO_2_).^42,43^ This silica coating induced a redshift of the longitudinal absorption peak to 775 nm (Figure 1b), thereby closely matching the 785 nm excitation wavelength and to maximizing Raman signal enhancement.

The morphology of the Au nanorods was examined using scanning electron microscopy (SEM) and bright-field transmission electron microscopy (TEM). As shown in Figure 2a,b, the nanorods exhibit an aspect ratio of approximately 3.3, with an average diameter of ∼ 30 nm and a length of ∼ 100 nm. Further structural characterization by dark-field TEM and energy-dispersive X-ray spectroscopy (EDX) (Figure S2) confirmed that the nanorods are composed exclusively of gold. High-resolution TEM (HR-TEM) images (Figure 2c) reveal well-defined atomic lattice fringes with a spacing of 2.38 Å, which can be indexed to the Au(111) facet. The corresponding fast Fourier transform (FFT) pattern (inset in Figure 2c) further corroborates this assignment through the presence of distinct diffraction spots characteristic of the (111) facet.

**Figure 2:**
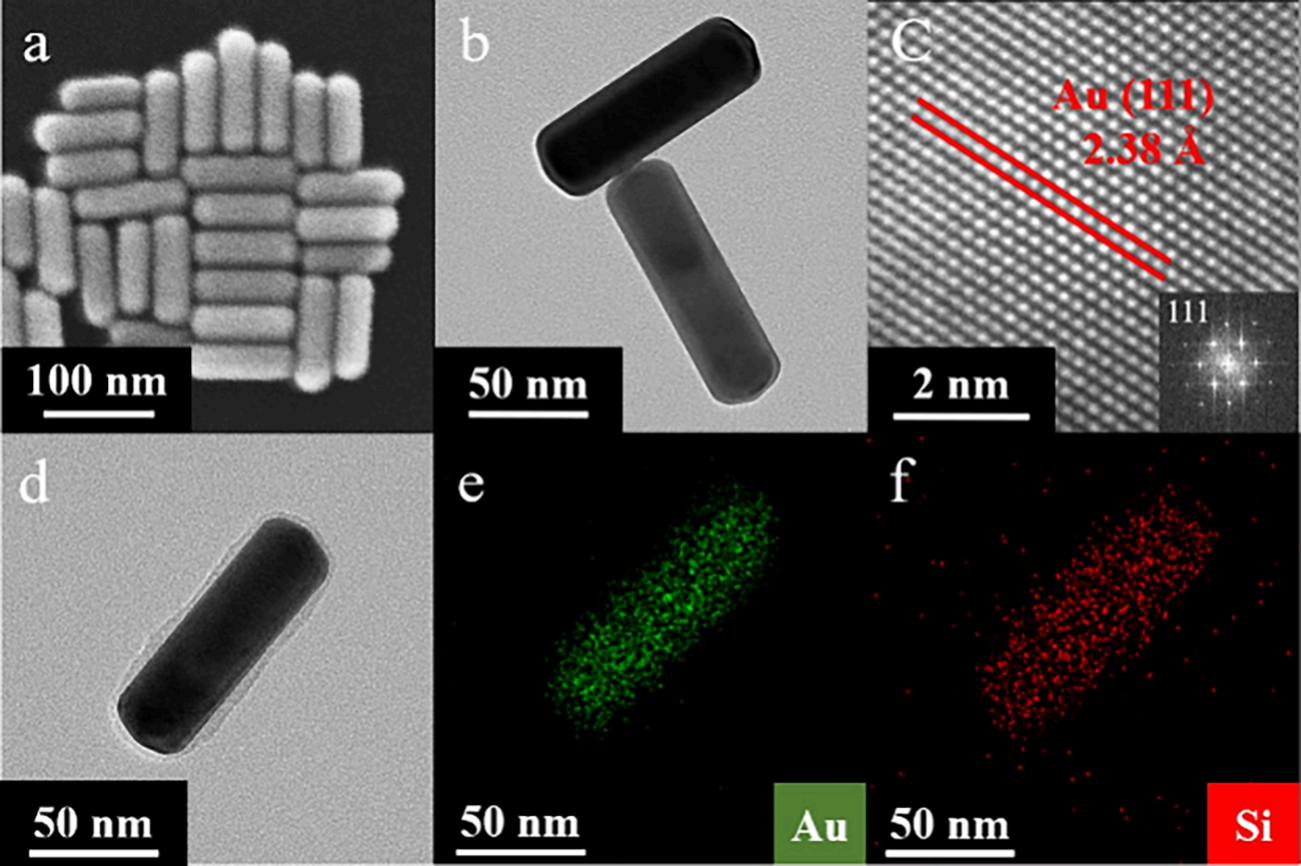
(a) SEM image of Au nanorods. (b) Bright-field TEM image of Au nanorods. (c) High-resolution TEM images of Au nanorods and the corresponding FFT pattern (inset). (d) Bright-field TEM image of AuNRs@SiO_2_. TEM EDX mapping of element (e) Au and (f) Si of AuNRs@SiO_2_.

Subsequently, a chemically inert silica shell was coated onto the surface of the Au nanorods to isolate the SERS-active nanostructures from direct interaction with the Au core. The structure of the resulting AuNRs@SiO_2_ was confirmed by bright-field and dark-field TEM, along with EDX (Figures 2d-f and S3). TEM analysis indicated that the Au nanorods retained their original morphology, thereby preserving their intrinsic optical properties. A uniform, pinhole-free SiO_2_ shell with an average thickness of approximately 5 nm was observed to fully encapsulate the Au nanorods. This coating induced a slight red-shift in the plasmonic absorption, which can be attributed to the increased longitudinal dimension. Line-scan EDX analysis (Figure S4) further confirmed the successful formation of the ∼ 5 nm silica shell surrounding the Au nanorods.

We integrated AuNRs@SiO_2_ into a custom-designed operando flow cell for enhanced mechanistic investigation. A benchmark metallic copper catalyst,^44–46^ whose metallic valence state and coordination environment were confirmed by X-ray absorption spectroscopy (Figure S5), was employed as the gas diffusion electrode (GDE). As shown in Figure S6, the customized operando flow cell exhibits current densities that closely match those of the electrochemical flow cell at the same applied potentials. In contrast, the operando H-cell shows significantly lower current densities under identical conditions. These results clearly demonstrate that the customized operando flow cell effectively replicates the catalytic environment of a practical flow-cell system, thereby enabling more representative operando measurements. The corresponding CO_2_RR performance is summarized in Table S2, demonstrating a C_2_ Faradaic efficiency of approximately 73% with suppressed hydrogen evolution below 10%, comparable to values reported in the literature (Table S3).^47–50^ Using this well-defined benchmark system, we subsequently reinvestigate the reaction intermediates involved in CO_2_ electroreduction on this benchmark copper catalyst under flow-cell operating conditions.

To systematically evaluate the effectiveness of flow-cell-compatible operando surface-enhanced Raman spectroscopy, we performed a series of comparative measurements, including (i) validation of the SERS enhancement effect using shell-isolated nanoparticles, and (ii) a combined comparison of conventional RCaman and SERS in the flow-cell configuration together with a parallel comparison between flow-cell and H-cell environments under otherwise comparable spectroscopic conditions.

For the successful implementation of operando SERS measurements under 785 nm excitation—selected to effectively suppress fluorescence background—the incorporation of AuNRs@SiO_2_ is critically necessary to provide sufficient electromagnetic enhancement without perturbing the catalytic system. To verify that the observed signal amplification originates from SERS enhancement effect rather than from catalytic modification, we synthesized Au nanoparticles and coated them with a thin SiO_2_ shell (AuNPs@SiO_2_).

SEM images (Figure S7) show that the as-prepared Au nanoparticles possess an average diameter of approximately 50 nm, and the SiO_2_ coating does not noticeably alter their size or dispersion. TEM bright-field imaging (Figure S8a) confirms their uniform morphology, while dark-field and EDX mapping analyses (Figure S8b–d) verify that the particles consist exclusively of Au. After coating, EDX elemental mapping and line-scan profiles (Figure S9) clearly demonstrate homogeneous encapsulation of the Au cores by a conformal SiO_2_ shell.

The UV–vis absorption spectrum (Figure S10) exhibits a surface plasmon resonance peak at ∼ 500 nm, which is well separated from the 785 nm excitation wavelength employed in Raman measurements. As a result, operando Raman spectra collected from AuNPs@SiO_2_-modified metallic Cu (Figure 3) are nearly identical to those obtained from bare Cu under identical electrochemical conditions, indicating that the presence of Au@SiO_2_ does not alter the reaction pathway or surface chemistry. Moreover, as shown in the Tables S4 and S5 and Figure S11, the incorporation of either AuNRs@SiO_2_ or AuNPs@SiO_2_ does not affect the catalytic activity or product distribution. Figure S12 shows that when pristine AuNRs or AuNPs are introduced, a reduction peak at approximately 0.83 V vs RHE is observed, which can be assigned to the reduction of Au^3+^,^51^ notably, this feature is absent for AuNRs@SiO_2_ and AuNPs@SiO_2_. The cathodic peaks observed at approximately 0.65, 0.4, and 0.2 V vs RHE can be attributed to the stepwise reduction of copper oxide species in alkaline media. Specifically, the peak at ∼ 0.65 V corresponds to the reduction of CuO/Cu­(OH)_2_ to Cu_2_O, while the peak at ∼ 0.4 V is assigned to the reduction of Cu_2_O to metallic Cu. The peak at ∼ 0.2 V is attributed to the reduction of residual oxide species remaining on the surface.^52–54^ These results indicate that the SiO_2_ shell effectively stabilizes the Au nanoparticles and prevents their participation in electrochemical processes. As further highlighted in Figure S13, no discernible microbubble formation was observed during spectral acquisition, and the gas diffusion electrode remained dry after the reaction. These observations collectively demonstrate the stability and effectiveness of the system. Repeated experiments using AuNRs@SiO_2_ for Raman signal enhancement consistently yielded similar results (Figure S14) demonstrating that the observed SERS enhancement effect is both reliable and reproducible. Collectively, these results confirm that the enhanced Raman signals arise from the electromagnetic amplification provided by the AuNRs@SiO_2_ configuration, rather than from any modification of the catalytic environment.

**Figure 3:**
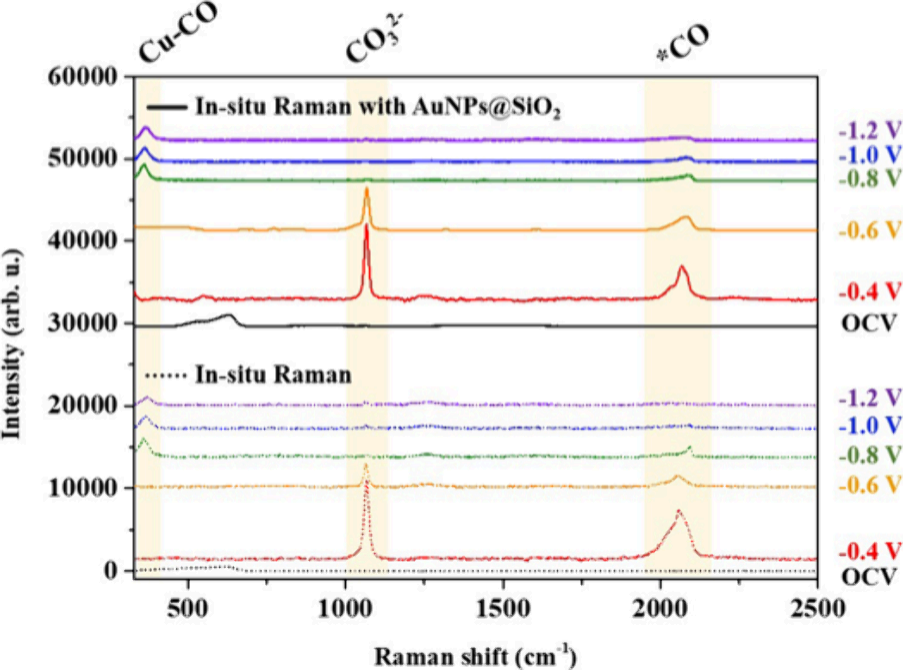
*Operando* Raman with and without AuNPs@SiO_2_ for benchmark sputtered copper recorded at various potentials during CO_2_RR in the flow cell.

We next conducted a combined comparison of conventional Raman and SERS measurements in the flow-cell configuration, together with a parallel comparison between flow-cell and H-cell environments under otherwise comparable spectroscopic conditions. Spectra were collected over a potential range of – 0.4 to – 1.2 V vs RHE (Figures 4 and S15–S16).

**Figure 4:**
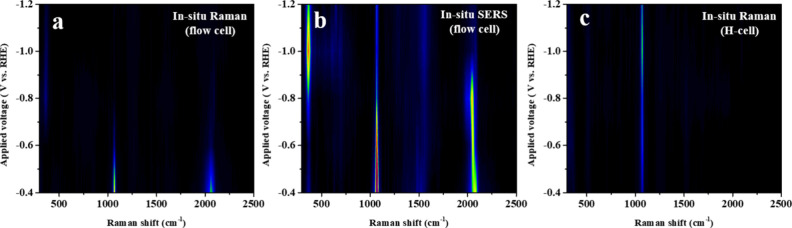
(a) *Operando* Raman spectra and (b) *operando* SERS spectral contours collected in a flow-cell configuration, and (c) *operando* SERS spectra contours acquired in a conventional H-cell, for the benchmark sputtered copper electrode at various applied potentials during CO_2_RR. The Raman intensity scales in (a) and (c) are aligned with that of (b) for direct comparison.

Under flow-cell conditions, conventional Raman spectroscopy (Figures 4a and S15) reveals characteristic intermediates commonly reported for Cu-based CO_2_RR catalysts.^55–57^ The band at 355 cm^–1^ corresponds to the interaction between adsorbed *CO and the Cu surface, indicating *CO adsorption on Cu active sites.^58–60^ The peak at 1060 cm^–1^ is assigned to the symmetric C–O stretching vibration of CO_3_^2–^ species,^61^ and its prominence, together with the absence of an HCO_3_^–^ signal, suggests the presence of a locally high-pH environment at the catalyst surface.^62^ The band at 2030 cm^–1^ is attributed to the C–O stretching vibration of adsorbed *CO.^63^

In contrast, SERS in the flow cell (Figures 4b and S15) provides substantially enhanced signal intensity and improved spectral resolution. In addition to amplifying the previously identified bands, SERS reveals additional features at approximately 700 and 1550 cm^–1^,^63^ which are assigned to the in-plane and asymmetric vibrational modes of the *CO_2_^–^ intermediate, respectively.^38^ A band at 1370 cm^–1^, corresponding to the *HOCCOH intermediate associated with C–C coupling,^36^ is also observed. These features are not observable using conventional Raman spectroscopy. On metal surfaces such as Au, Cu, and Ag, which typically operate at medium to high overpotentials, *CO_2_^–^ is commonly recognized as the first intermediate formed during CO_2_ reduction.^64–66^ Thus, the activation of the stable CO_2_ can be successfully observed through SERS technique in the flow cell configuration.

*HOCCOH, on the other hand, is a critical intermediate associated with the C–C coupling of adsorbed *CO species.^67,68^ These findings highlight the superior sensitivity of the SERS technique in capturing subtle yet critical intermediates that may otherwise be missed using traditional Raman approaches.

Finally, to assess the influence of the reaction environment, we compared *operando* SER measurements performed in a customized H-cell (Figures 4c and S16) with those obtained in the flow-cell configuration. In the H-cell, where CO_2_ solubility and mass transport are limited,^11,69^ only weak bands corresponding to surface-bound *OH (∼520 cm^–1^) and CO_3_^2–^ (∼1060 cm^–1^) are observed, with no detectable *CO intermediates. This observation suggests that the H-cell provides a markedly different catalytic microenvironment from that of a flow cell, resulting in sluggish CO_2_RR kinetics and potentially a distinct reaction pathway. In addition, the low laser power used for in situ Raman measurements to prevent thermal damage to the PTFE substrate may also contribute to the weak CO* signal intensity. Taken together, these factors explain why *CO intermediates were not detected in the operando H-cell. In contrast, the flow-cell spectra display rich intermediate features, including *CO, *CO_2_^–^, and *HOCCOH species, consistent with enhanced CO_2_ mass transport and higher catalytic turnover. Notably, benefiting from the significant signal enhancement in the flow-cell-compatible operando surface-enhanced Raman spectroscopy (SERS) platform, the broad *CO band near 2030 cm^–1^ in Figure 5a can be deconvoluted into three distinct components: bridge-bonded *CO (CO_bridge_, ∼ 2000 cm^–1^), low-frequency band CO (LFB-CO, ∼ 2050 cm^–1^), and high-frequency band CO (HFB-CO, ∼ 2090 cm^–1^).^19,70,71^ Meanwhile, both the bridged CO and LFB-CO peaks display systematic shifts toward lower Raman wavenumbers with increasing negative potentials. The bridged CO peak shifts from 2014 cm^–1^ at – 0.4 V to 1995 cm^–1^ at – 1.0 V, while the LFB-CO peak shifts from 2055 cm^–1^ to approximately 2045 cm^–1^ over the same potential range. These shifts can be attributed to the electrochemical Stark effect,^38^ arising from the interaction between the interfacial electric field and the adsorbed CO species, leading to a potential-dependent modulation of the C≡O vibrational frequency. Previous studies have suggested that LFB-CO, characterized by a weakened C≡O bond and closer adsorbate proximity,^72–74^ is more reactive toward C–C coupling and ethylene formation, whereas HFB-CO is more likely to desorb as CO.^73^ The LFB-CO signal is significantly intensified in the flow-cell configuration, which correlates with the predominant formation of ethylene, as evidenced by the high Faradaic efficiency of approximately 50% achieved with the benchmark metallic copper catalyst.

**Figure 5:**
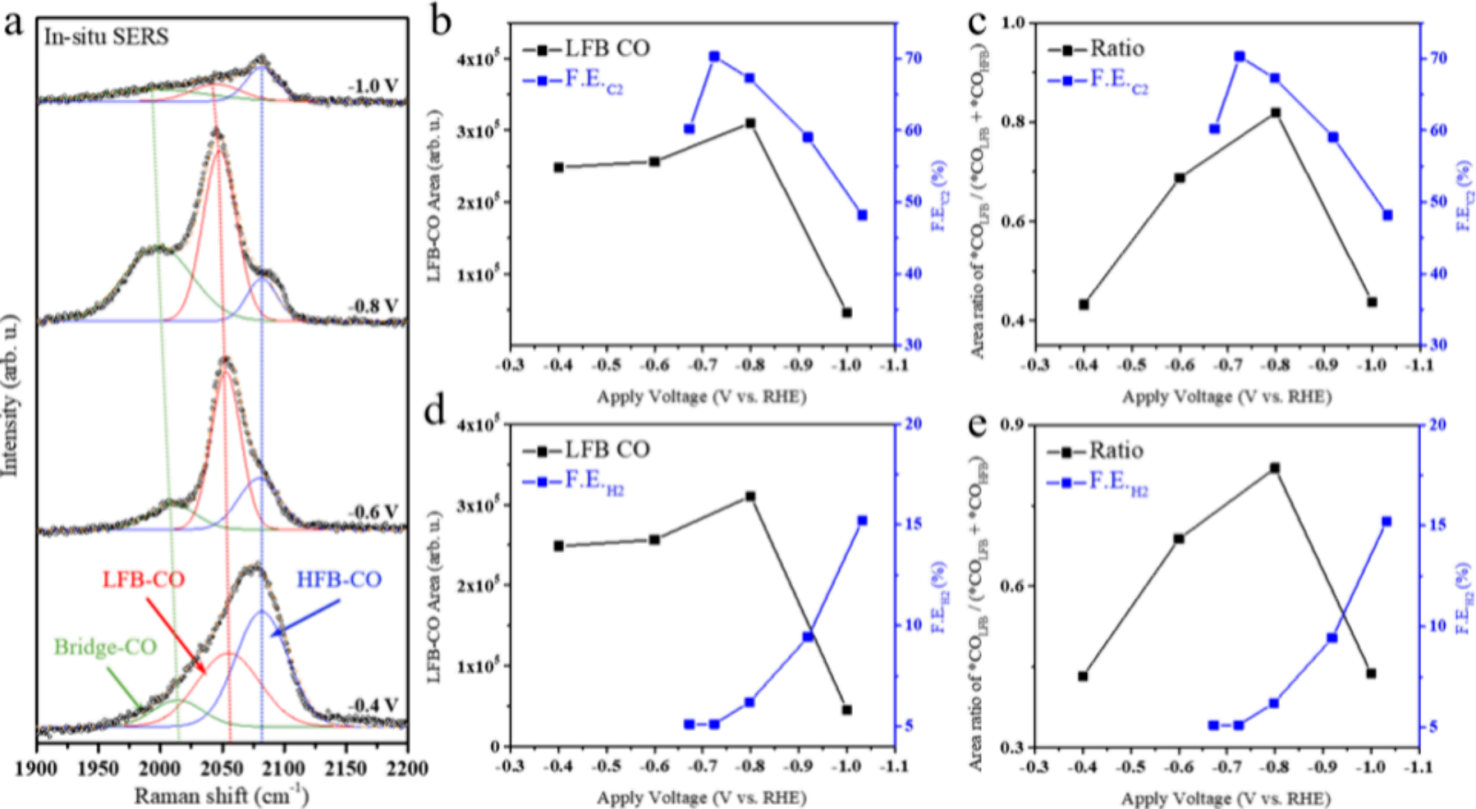
(a) In-situ SERS showing CO* for benchmark sputtered copper recorded at various potentials during CO_2_RR in a flow cell. (b) LFB CO area and F.E._C2_ as a function of applied voltage in a flow cell. (c) Ratio of LFB CO/(LFB CO + HFB CO) and F.E._C2_ as a function of applied voltage in a flow cell. (d) LFB CO area and F.E._H2_ as a function of applied voltage in a flow cell. (e) Ratio of LFB CO/(LFB CO + HFB CO) and F.E._H2_ as a function of applied voltage in a flow cell.

Quantitative analysis reveals a strong correlation between LFB-CO population and C_2_ product selectivity. As the applied potential becomes more negative, the integrated LFB-CO peak area initially increases and then decreases, with maximum intensity observed between – 0.7 and – 0.8 V, coinciding with the highest C_2_ Faradaic efficiency (Figure 5b and Table S2). The ratio LFB/(LFB + HFB) increases nearly linearly from – 0.4 to – 0.7 V (Figure 5c), closely tracking the evolution of C_2_ selectivity in this region. We attribute this behavior to the relatively larger contribution of HFB-CO at less negative potentials. Since HFB-CO has been reported to preferentially desorb as CO (Figure S17 shows HFB-CO area and F.E._CO_ with positive correlation),^70,73,75^ considering both HFB-CO and LFB-CO populations provides a more comprehensive description of how surface-bound *CO governs C_2_ selectivity, particularly at low overpotentials. At more negative potentials, a pronounced decrease in LFB-CO population and in the LFB/(LFB + HFB) ratio correlates with a significant increase in H_2_ Faradaic efficiency (Figures 5d,e), suggesting that insufficient *CO coverage under strong driving force promotes a shift toward hydrogen evolution. Because C–C coupling requires overcoming a relatively high energy barrier, lower applied potentials lead to sluggish C–C coupling kinetics, and the corresponding F.E._C2_ is mainly dictated by the applied potential.^76^ Once the potential exceeds – 0.8 V vs RHE, a larger fraction of CO* intermediates can overcome this barrier. Given that C–C coupling proceeds through the interaction of two adjacent CO* species, considering the proportion of LFB-CO within the total atop-CO population, namely its surface concentration, provides a more accurate correlation with F.E._C2_. These findings demonstrate that SERS enables direct correlation between surface-bound *CO speciation and product distribution under realistic high-current-density conditions.

Collectively, these results demonstrate that the combination of a flow-cell configuration and SERS enhancement is essential for capturing transient reaction intermediates under practically relevant CO_2_RR conditions. This approach provides improved sensitivity, spectral resolution, and mechanistic insight into C–C coupling and product selectivity that cannot be achieved using conventional Raman spectroscopy or H-cell configurations alone.

In conclusion, we have established a flow-cell-compatible operando SERS platform by integrating silica-coated Au nanorods (AuNRs@SiO_2_) optimized for 785 nm excitation. The plasmon resonance was tuned to maximize electromagnetic enhancement while suppressing fluorescence, and the inert SiO_2_ shell ensured that signal amplification occurred without perturbing the catalytic surface. Combined comparisons between conventional Raman and SERS in a flow cell, together with parallel measurements in a conventional H-cell, demonstrate the necessity of both plasmonic enhancement and realistic mass-transport conditions. While conventional Raman detects only dominant species such as *CO and CO_3_^2–^, *operando* SERS reveals additional key intermediates, including *CO_2_^–^ and *HOCCOH, directly associated with CO_2_ activation and C–C coupling. Deconvolution of the *CO band further enables correlation between surface-bound CO speciation and C_2_ selectivity. These results highlight that integrating flow-cell operation with operando SERS is essential for capturing transient intermediates and establishing mechanistic insight under practically relevant high-rate CO_2_RR conditions.

## Supplementary Material



## Data Availability

The data supporting this study are available in the paper and the Supporting Information. All other relevant source data are available from the corresponding authors upon reasonable request.

## Notes

Experimental Section, normalized absorption spectrum, SEM images and TEM images of AuNRs@SiO_2_ nanorods and shell-isolated Au nanoparticles, electrochemical properties and CO_2_RR performances of the electrocatalysts, and additional operando Raman spectra (PDF)
