# Supplementary material for: Flow-Cell-Compatible Operando Surface-Enhanced Raman Spectroscopy for Probing Reaction Intermediates during Carbon Dioxide Reduction Reaction
Source: J Phys Chem Lett. 2026 May 19;17(26):7379–86. doi: 10.1021/acs.jpclett.6c00902 (PMC13339785; doi:10.1021/acs.jpclett.6c00902)
Supplement: Supplementary file 1 [file jz6c00902_si_001.pdf]

Supporting Information

Flow-Cell-Compatible Operando Surface-Enhanced Raman Spectroscopy for Probing Reaction Intermediates during Carbon Dioxide Reduction Reaction

Yu-Jhih Shen,[1] Yung-Hsi Hsu,[1] Yu-Chia Chang,[1] Yu-Cheng Liu,[1] Kang-Shun Peng,[1] Chih-Wei Hu,[3] Ying-Rui Lu,[3] Shao-Hui Hsu,[4] Sung-Fu Hung[1,2*]

[1]*Department of Applied Chemistry and Center for Emergent Functional Matter Science, National Yang Ming Chiao Tung University, Hsinchu 300, Taiwan.*
[2]*Department of Medicinal and Applied Chemistry, Kaohsiung Medical University, Kaohsiung 807, Taiwan.*
[3]*National Synchrotron Radiation Research Center, Hsinchu 300, Taiwan*
[4]*Taiwan Semiconductor Research Institute, National Applied Research Laboratories, Hsinchu 300, Taiwan.*

Email: sungfuhung@nycu.edu.tw

This file includes:

Experimental Section

Supplementary Figures 1 to 17.

Supplementary Tables 1 to 5.

Experimental Section

Chemicals

All chemical were obtained from commercial suppliers and used without further purification. Hexadecyltrimethylammonium bromide (CTAB, > 98.0%) was purchased from Alfa Aesar, sodium oleate (NaOL, > 97.0%) was purchased from Angene, hydrogen tetrachloroaurate trihydrate ($HAuCl_4 \cdot 3H_2O$) was purchased from Alfa Aesar. L-ascorbic Acid (> 99.7%) was purchased from Honeywell, silver nitrate ($AgNO_3$, > 99%) was purchased from Honeywell, sodium borohydride ($NaBH_4$, 99%) was purchased from Thermo Scientific and hydrochloric acid (HCl, 37 wt% in water) were purchased from Sigma Aldrich. (3-Aminopropyl)trimethoxysilane (APTMs, 98%) was purchased from ThermoFisher Scientific, sodium silicate solution (≥27% SiO_2 basis) and Nafion solution (5% in lower aliphatic alcohols and water, contains 15–20% water).

Synthesis of Gold Nanorods (AuNRs)

The seed solution for gold nanorods (NRs) growth was prepared by mixing 5 mL of 0.5 mM $HAuCl_4$ with 5 mL of 0.2 M CTAB solution,[1] followed by rapid injection of 1 mL of freshly prepared 0.006 M $NaBH_4$ under vigorous stirring (1200 rpm). Stirring was stopped after 2 min, and the seed solution was aged at 30 °C for 30 min prior to use.
For preparation of the growth solution, 700 mg of CTAB and 123 mg of NaOL were dissolved in 25 mL of warm water (~50 °C). After cooling to 30 °C, 2.4 mL of 4 mM $AgNO_3$ solution was added, and the mixture was left undisturbed at 30 °C for 15 min. Subsequently, 25 mL of 1 mM $HAuCl_4$ was introduced. The solution gradually became colorless after stirring at 700 rpm for 90 min, after which 0.21 mL of concentrated HCl (37 wt. % in water, 12.1 M) was added. Following an additional 15 min of slow stirring at 400 rpm, 0.125 mL of 0.064 M ascorbic acid (AA) was added, and the solution was vigorously stirred for 30 s. Finally, 0.04 mL of the seed solution was injected into the growth solution, followed by stirring for 30 s. The reaction mixture was then left undisturbed at 30°C for 12 h for gold nanorods growth. The resulting gold nanorods were collected by centrifugation and finally redispersed in 20 mL of deionized water.

Preparation of SiO_2 Shell-Isolated Gold Nanorods (AuNRs@SiO_2)

100 μL of fresh prepared 1 mM APTMs was added to 10 mL of an Au nanorods dispersion under vigorous stirring (1200 rpm) for 15 min.[2] Subsequently, 800 μL of freshly prepared sodium silicate solution (dilute to 0.54 wt. % with deionized water and acidified to pH ~10.2 using hydrochloric acid) was added at room temperature, followed by vigorous stirring (1200 rpm) for an additional 30 min. The resulting products were collected by centrifugation and finally redispersed in 1 mL of deionized water.

Synthesis of Gold Nanoparticles (AuNPs)

Gold nanoparticles with an average diameter of approximately 55 nm were synthesized using a citrate reduction method. In a typical procedure, an aqueous solution of chloroauric acid (200 mL, 0.01 wt%) was heated to boiling under vigorous stirring and reflux. A sodium citrate solution (1.4 mL, 1 wt%) was then rapidly injected into the boiling solution, and the reaction was maintained at reflux for 30 min. After cooling to room temperature, the resulting Au nanoparticles were collected by centrifugation and finally redispersed in 200 mL of deionized water.

Preparation of SiO_2 Shell-Isolated Gold Nanoparticles (AuNPs@SiO_2)

Au@SiO_2 shell-isolated gold nanoparticles (AuNPs@SiO_2) were prepared as follows. A total of 30 mL of the as-prepared Au nanoparticle solution was mixed with 0.4 mL of 1 mM APTMS and stirred for 15 min. Subsequently, 3.2 mL of sodium silicate solution (diluted to 0.54 wt% with deionized water and adjusted to pH ≈ 10.2 using hydrochloric acid) was added, and mixture was stirred for an additional 3 min at room temperature. The reaction mixture was then heated at 90 °C for 30 min to enable controlled growth of the SiO_2 shell. The reaction was quenched by transferring 1.5 mL aliquots into ice-cooled centrifuge tubes. The resulting SiO_2 shell-isolated Au nanoparticles were collected by centrifugation.

Characterization

The structural morphology was characterized using a field-emission scanning electron microscope (FESEM, JEOL, JSM-6700F). High-resolution lattice fringes and elemental mapping were acquired using a field-emission transmission electron microscope (FE-TEM, JEOL-2100F) equipped with an energy-dispersive X-ray spectroscopy (EDX) detector (Oxford Instrument XMaxN TSR) at the Department of Chemistry, National Taiwan University, Taiwan. X-ray absorption spectroscopy (XAS), including X-ray absorption near-edge spectra (XANES) and extended X-ray absorption fine structure (EXAFS) measurements at the Cu K-edge, was conducted in fluorescence mode at the BL32A beamline of TPS, NSRRC. The pre-edge baseline was subtracted, and the spectra were normalized to the post-edge region. EXAFS analysis was performed by applying a Fourier transform to the k^2-weighted EXAFS oscillations to evaluate the contributions of individual atomic coordination shells to the corresponding Fourier transform peaks.

Preparation of a Benchmark Copper Gas-Diffusion Electrode

For the benchmark copper gas-diffusion electrode, a 300 nm thick copper was deposited onto a PTFE substrate by magnetron sputtering using a high-purity Cu target

(99.99%). The sputtering was performed under an Ar atmosphere at a working pressure of 3 mTorr with a DC power of 30 W. The base pressure of the sputtering chamber was maintained below 5×10^{-6} Torr using a turbomolecular pump.

Electrochemical measurement

The electrochemical properties were examined using a Biologic VSP-3e potentiostat in a flow cell reactor, where a gas diffusion electrode (GDE), nickel foam, and a saturated Ag/AgCl electrode were served as the working electrode (WE), counter electrode (CE), and reference electrode (RE), respectively. The WE and CE were separated by an anion exchange membrane. CO_2 was supplied at a flow rate of 50 sccm. All potentials were converted to the reversible hydrogen electrode (RHE) scale using the equation: $E_{RHE} = E_{Ag/AgCl} + 0.0591 \times pH + E^0_{Ag/AgCl}$, where $E^0_{Ag/AgCl}$ (0.210 V) is the standard potential of Ag/AgCl relative to the standard hydrogen electrode (SHE) at 25 °C. The potentials were further corrected by iR compensation. Gaseous products were analyzed using gas chromatography (Agilent 8860) equipped with a thermal conductivity detector and a flame ionization detector, and were quantified using calibration curves established for each gas product. Liquid products were analyzed using high performance liquid chromatography (Agilent 1260 Infinity II) equipped with a variable wavelength detector and a refractive index detector for HCOOH, as well as gas chromatography-mass spectrometry (Agilent 7860/5975C GC/MS) for C_2H_5OH and C_3H_7OH. The liquid products were quantified using calibration curves established for each liquid product.

***Operando* Surface-Enhanced Raman Spectroscopy in an Operando Flow Cell**

AuNRs@SiO_2, prepared by adding 4 μL Nafion solution (~5 wt. %) into 100 μL AuNRs@SiO_2 solution, was sprayed onto the gas-diffusion electrode. This gas-diffusion electrode was assembled in a custom-designed operando flow cell, where a gas diffusion electrode (GDE) served as the working electrode (WE), nickel foam as the counter electrode (CE), and a saturated Ag/AgCl electrode as the reference electrode (RE). The WE and CE were separated by an anion exchange membrane. Raman spectra were performed using a Renishaw inVia Raman microscope equipped with a water immersion objective and a 785 nm laser source with a maximum output power of 320 mW. To prevent thermal damage to the PTFE substrate, which is susceptible to degradation under intense laser irradiation, the laser power was set to 0.1% of the maximum output, corresponding to approximately 0.32 mW at the sample surface. Each spectrum was acquired with an integration time of 10 s and one accumulation. During *operando* Raman measurements, CO_2 gas was continuously supplied to the gas chamber at a flow rate of 50 sccm.

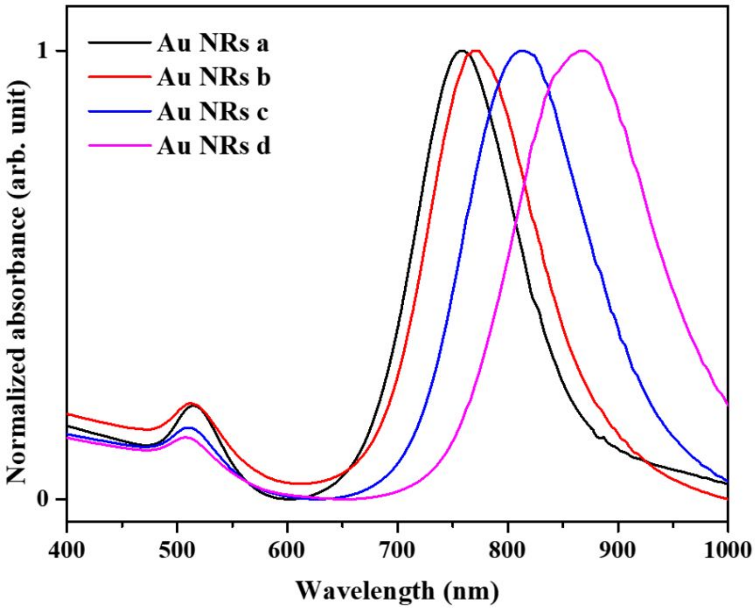

Figure S1. Normalized absorption spectrum of the synthesized Au nanorods, whose synthetic parameters are listed in Table S1.

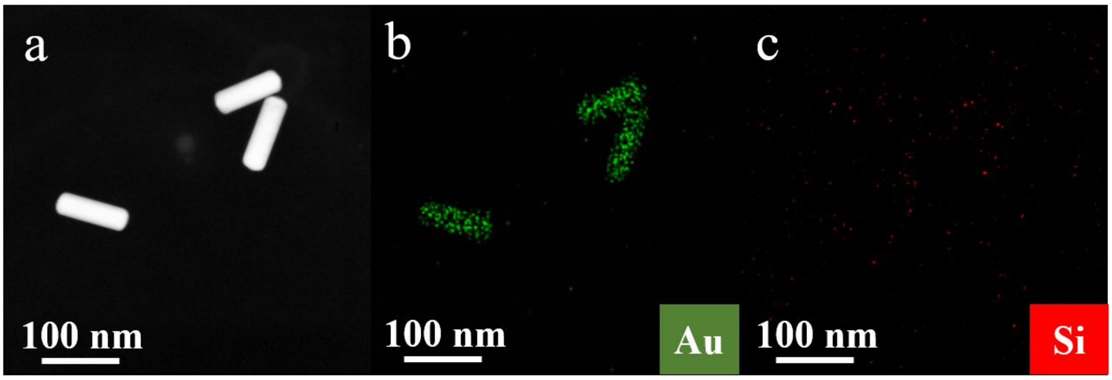

Figure S2. TEM images of Au nanorods in (a) dark field and EDX mapping of element (b) Au and (c) Si.

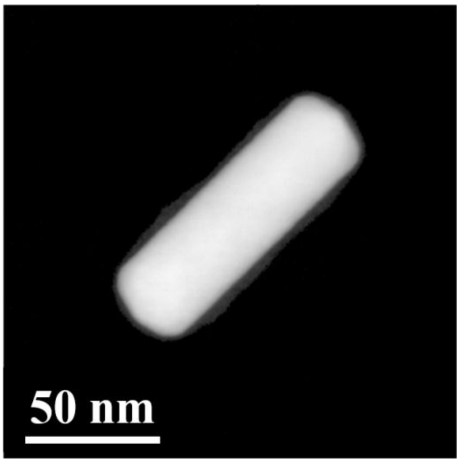

Figure S3. Dark field TEM image of AuNRs@SiO_2.

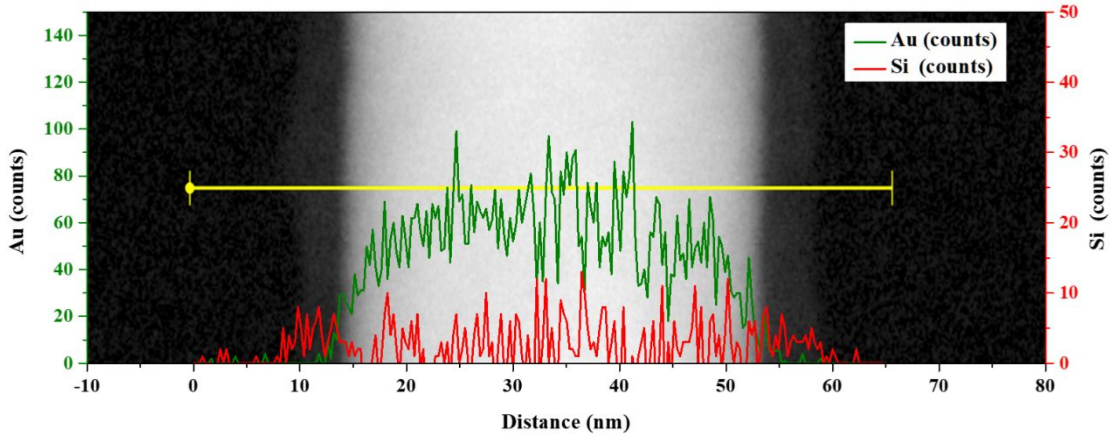

Figure S4. TEM images of AuNRs@SiO_2 nanorods in dark field and EDX line mapping of element Au and Si.

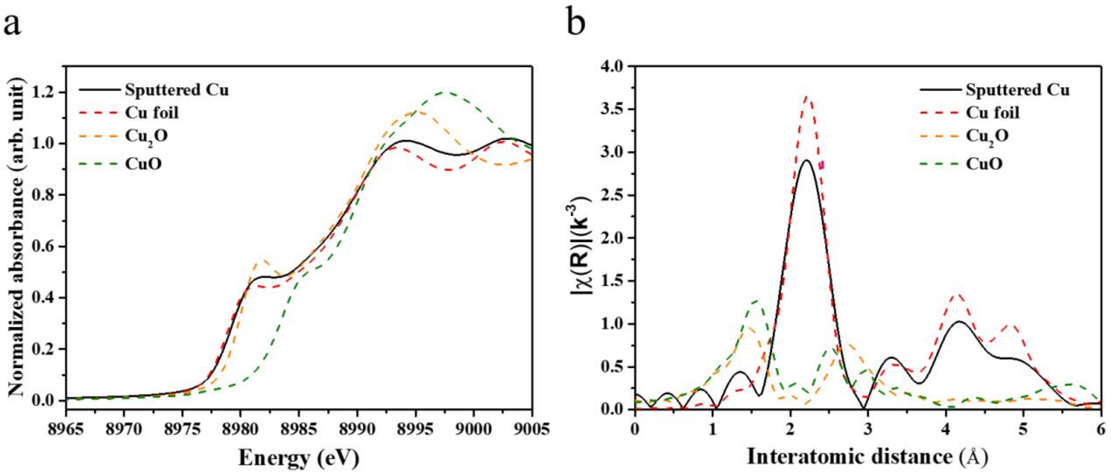

Figure S5. (a) XANES of sputtered Cu and (b) EXAFS of sputtered Cu.

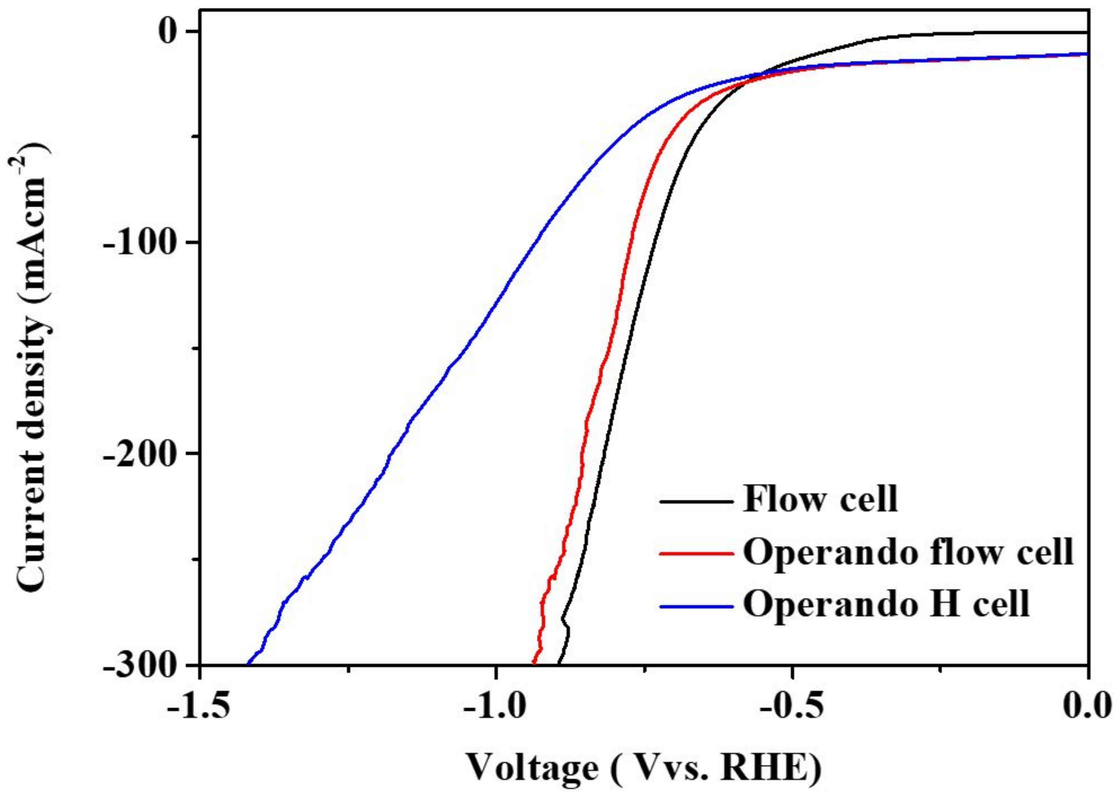

Figure S6. LSV curve of benchmark metallic Cu measured in flow cell, operando flow cell and operando H cell.

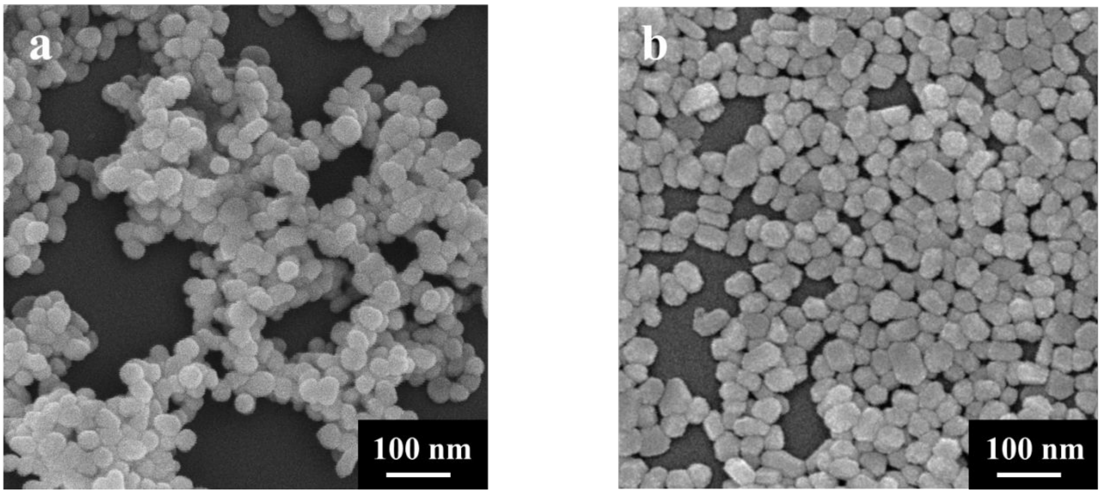

Figure S7. SEM image of (a) Au nanoparticles and (b) AuNPs@SiO_2.

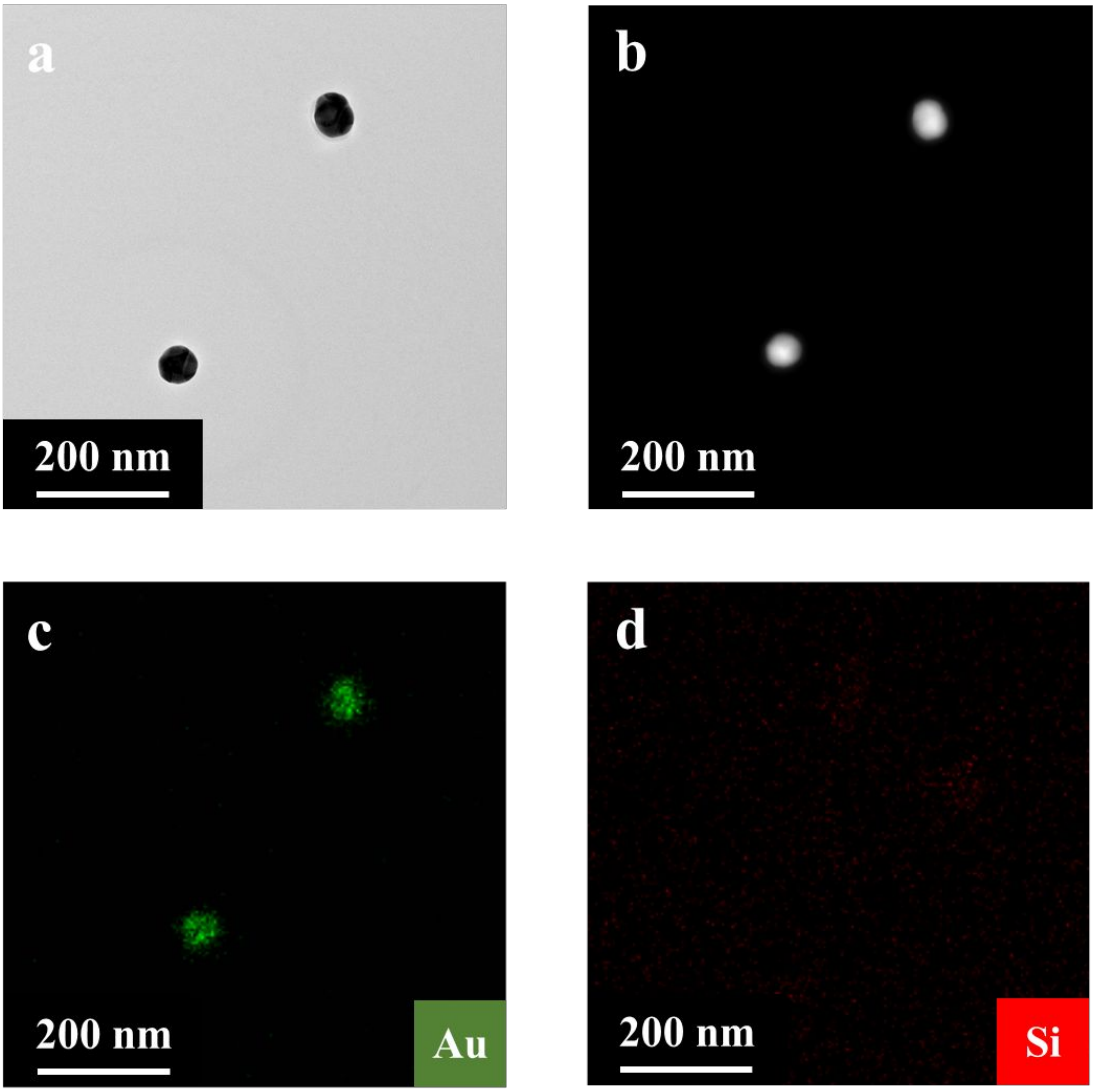

Figure S8. (a) Bright-field TEM image of Au nanoparticles. (b) Dark-field TEM image of Au nanoparticles. Au nanoparticles EDX mapping of element (c) Au and (d) Si.

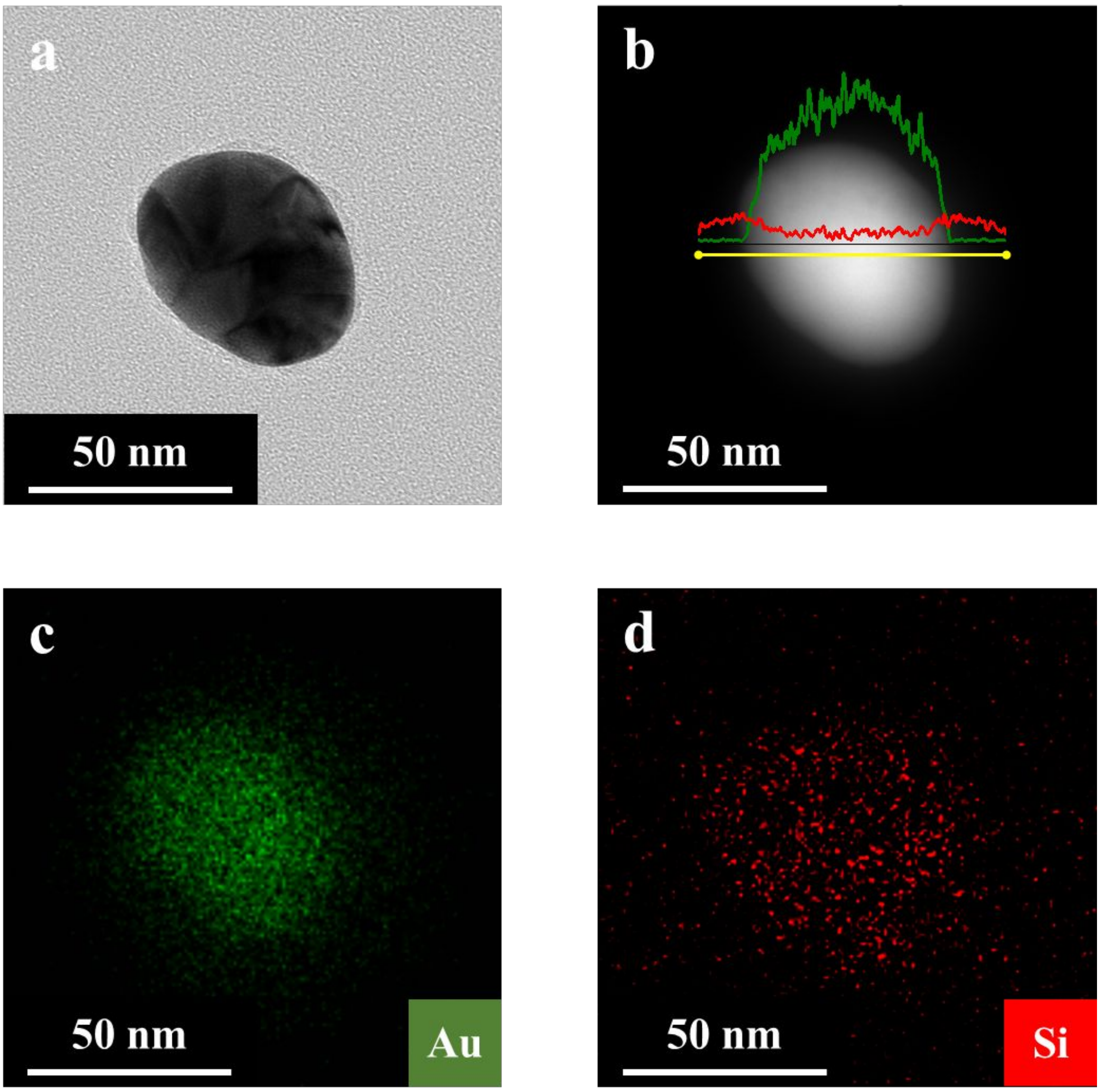

Figure S9. (a) Bright-field TEM image of AuNPs@SiO_2. (b) Dark-field TEM image and line scan of Au nanoparticles (Au: green line, Si: red line). AuNPs@SiO_2 EDX mapping of element (c) Au and (d) Si.

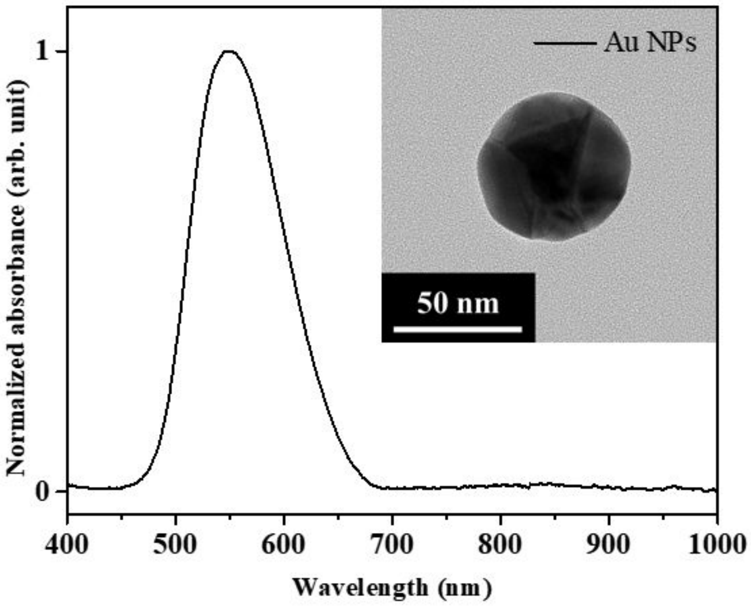

Figure S10. Normalized absorption spectrum and TEM image of shell-isolated Au nanoparticles.

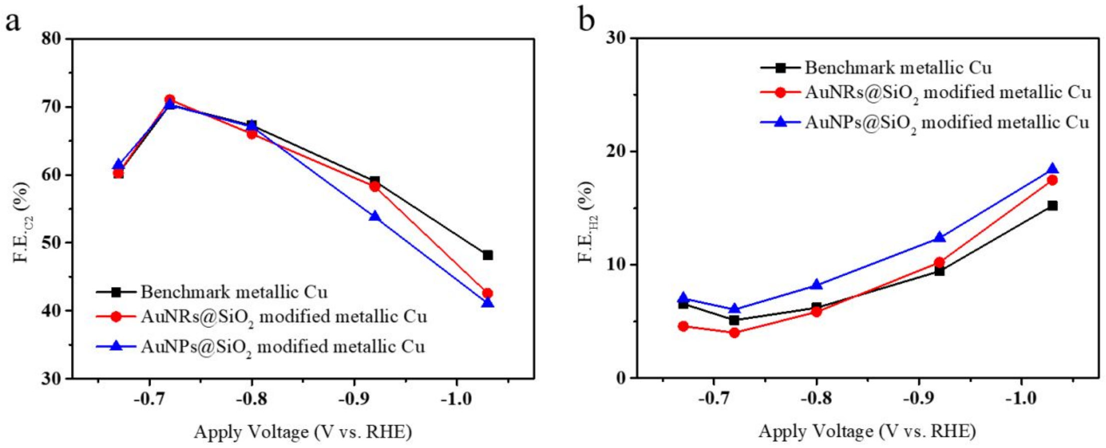

Figure S11. (a) F.E.$_{C2}$ as a function of applied voltage of benchmark metallic Cu, AuNRs@SiO_2 modified metallic Cu and AuNPs@SiO_2 modified metallic Cu. (b) F.E.$_{H2}$ as a function of applied voltage of benchmark metallic Cu, AuNRs@SiO_2 modified metallic Cu and AuNPs@SiO_2 modified metallic Cu.

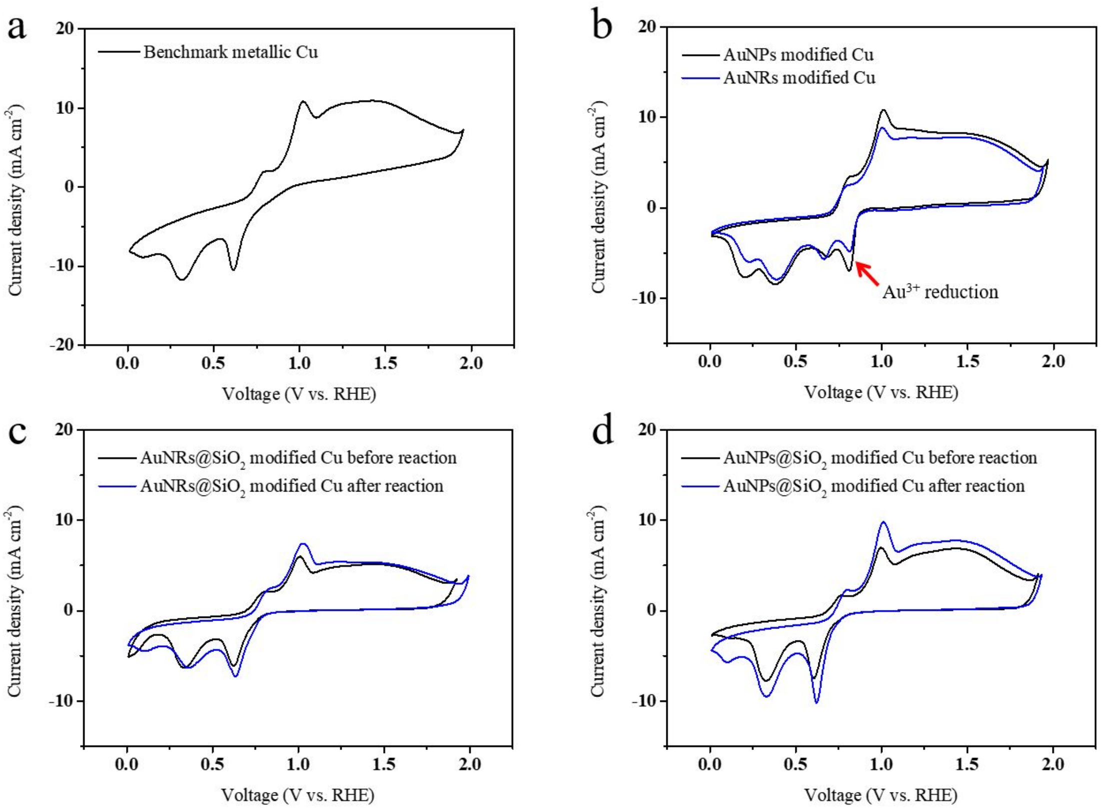

Figure S12. CV curve of (a) benchmark metallic Cu, (b) AuNPs, AuNRs modified Cu, (c) AuNRs@SiO_2 modified Cu before and after reaction, and (d) AuNPs@SiO_2 modified Cu before and after reaction.

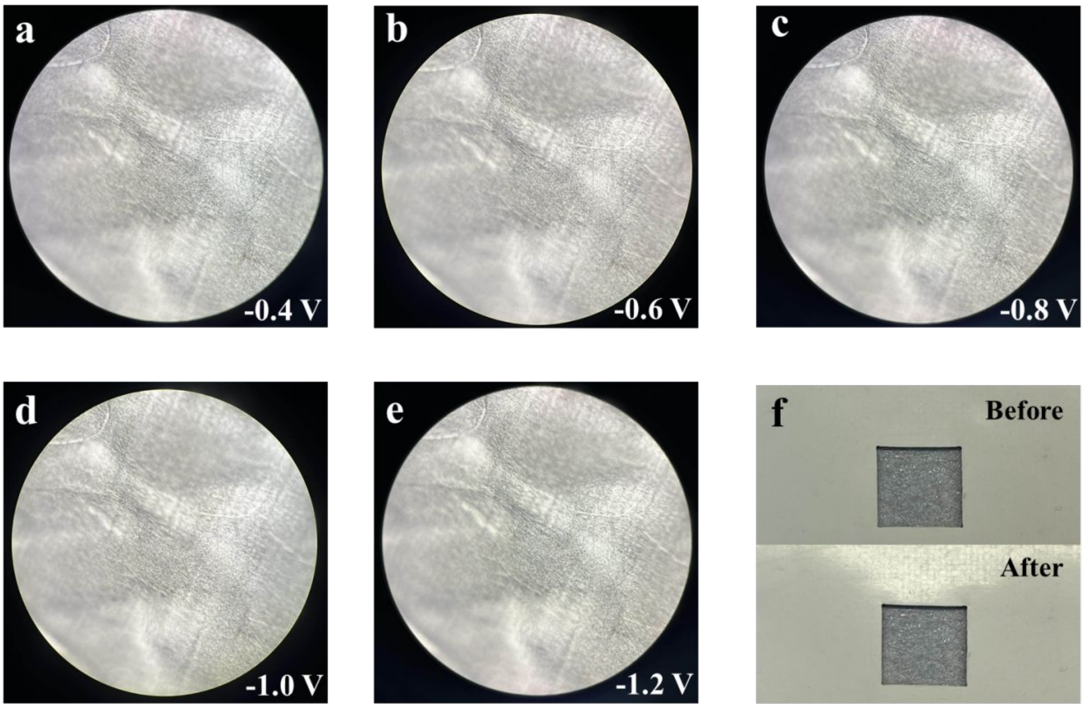

Figure S13. Microscope image capture during applied voltage of (a) -0.4, (b) -0.6, (c) -0.8, (d) -1.0 and (e) -1.2 V vs. RHE. (f) Backside of image GDE before and after reaction.

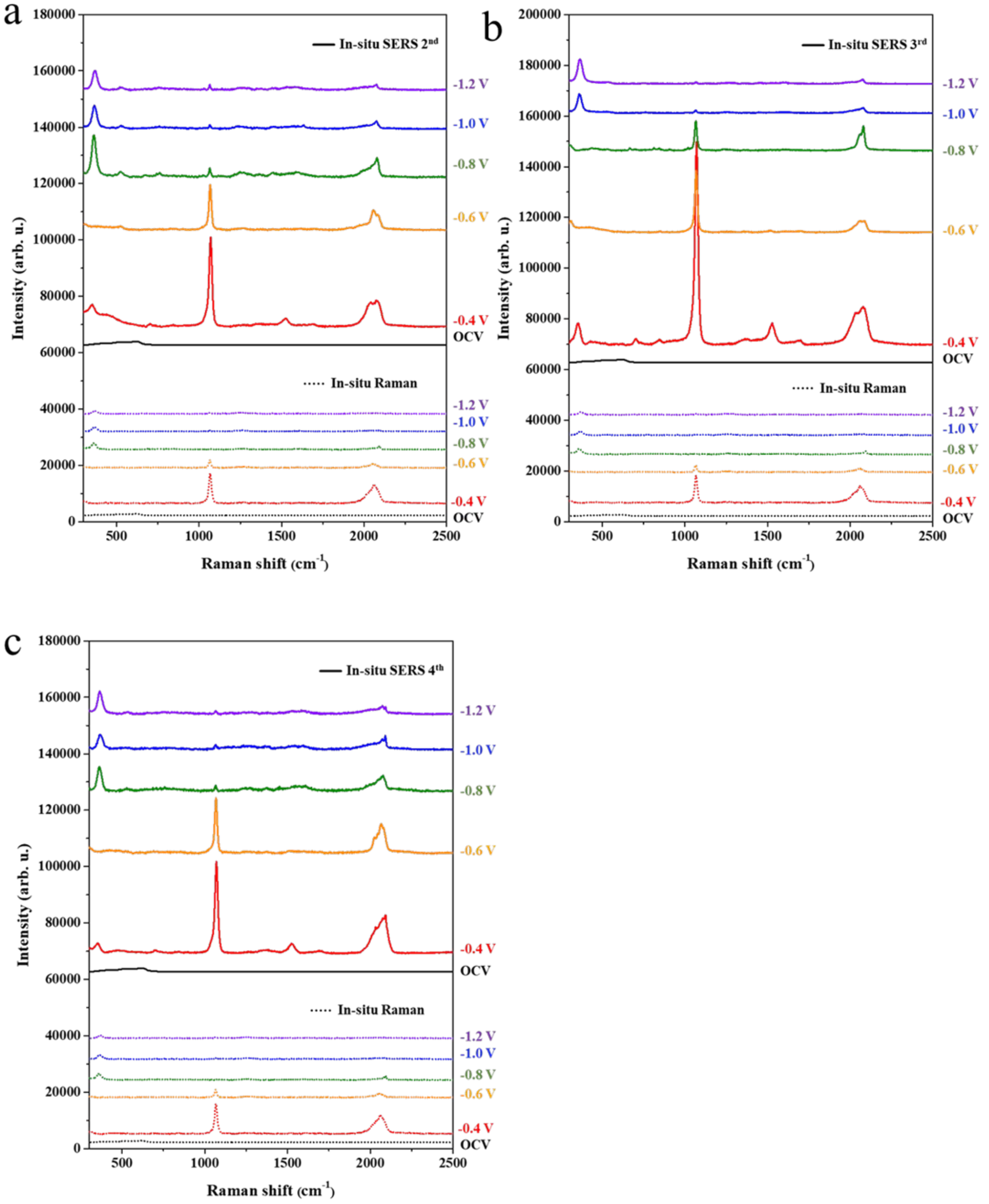

Figure S14. Reproduce experiments of in-situ SERS for the (a) first, (b) second and (c) third time.

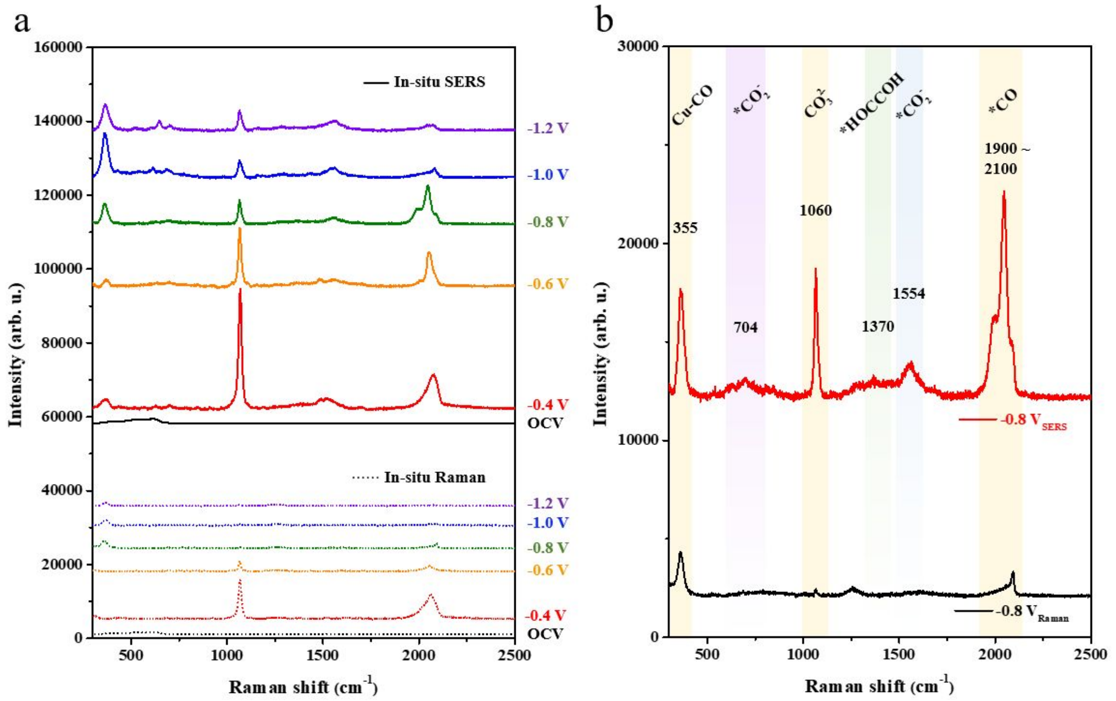

Figure S15. (a) *Operando* Raman and *Operando* SERS for benchmark sputtered copper recorded at various potentials during CO_2RR. (b) In-situ spectra of Raman and SERS collect at -0.8 V vs. RHE.

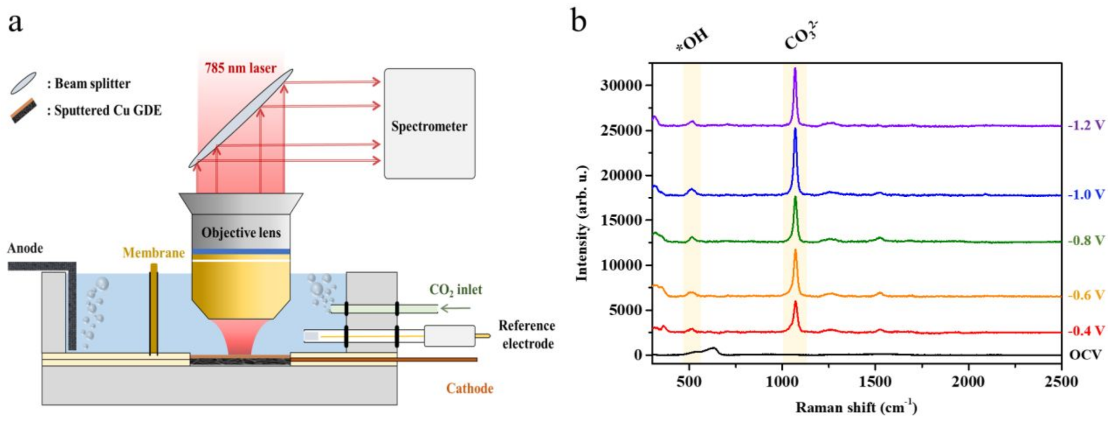

Figure S16. (a) Schematic of in-situ SERS spectroscopy under H-cell environment. (b) In-situ SERS for benchmark sputtered copper recorded at various potentials during CO_2RR under H-cell environment.

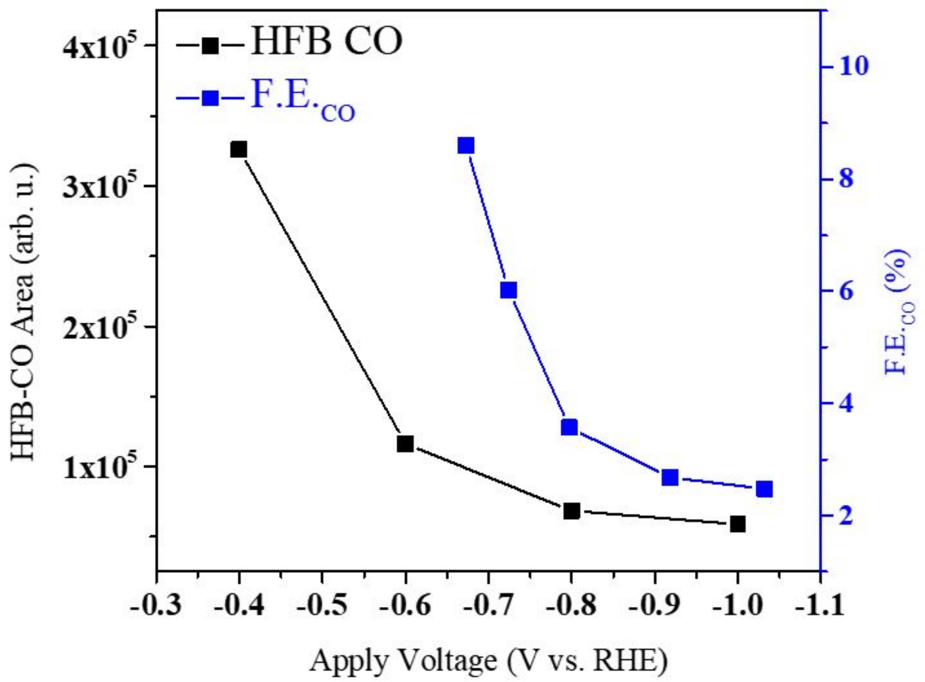

Figure S17. HFB CO area and F.E.$_{CO}$ as a function of applied voltage in a flow cell.

Table S1. Synthetic parameters of the Au nanorods in this study

**Sample**	**CTAB (mg)**	**$AgNO_3$ (mL)**	**Seed (mL)**	**HCl (mL)**	**NaOL (mg)**	**SPR (nm)**
Au NRs a	700	1.8	0.04	0.15	123	758
Au NRs b	700	2.4	0.04	0.21	123	770
Au NRs c	700	2.4	0.08	0.21	123	813
Au NRs d	700	1.8	0.08	0.21	123	866

Table S2. Faradaic efficiencies of CO_2RR products at various applied voltage for benchmark metallic copper

Sample	Voltage (vs RHE)	FE_{CO} (%)	FE_{C2H4} (%)	FE_{CH4} (%)	FE_{H2} (%)	FE_{HCOOH} (%)	FE_{C2H5OH} (%)	FE_{C3H7OH} (%)
Benchmark metallic Cu	-0.67	8.60	44.39	N.A.	6.55	8.31	15.82	N.A.
	-0.72	6.02	50.09	N.A.	5.12	5.52	20.21	2.51
	-0.80	3.57	47.67	4.45	6.2	4.73	19.58	N.A.
	-0.92	2.68	41.76	8.15	9.43	4.39	17.29	N.A.
	-1.03	2.48	33.26	14.18	15.21	4.42	14.93	N.A.

Table S3. Comparison of Faradaic efficiencies of C_2 products for benchmark metallic copper in the literature.

Sample	Electrolyte	Highest FE_{C2} (%)	Reference
Benchmark metallic Cu	1 M KOH	72.81	This work
Sputtered Cu	1 M KOH	70	*Mater. Today Sustain.* **2025**, *30*, 101124.
EB-Cu	1 M KOH	70	*ACS Appl. Mater. Interfaces.* **2022**, *14* (6), 7731-7740.
Cu_{300}	1 M KOH	78.76	*Energy & Fuels* **2026**, *40* (1), 555-564.
Bare Cu	0.5 M $KHCO_3$	66.8	*Green Chem.* **2025**, *27* (21), 6039-6055.

Table S4. Faradaic efficiencies of CO_2RR products at various applied voltage for AuNRs@SiO_2 modified metallic Cu.

Sample	Voltage (vs RHE)	FE_{CO} (%)	FE_{C2H4} (%)	FE_{CH4} (%)	FE_{H2} (%)	FE_{HCOOH} (%)	FE_{C2H5OH} (%)	FE_{C3H7OH} (%)
AuNRs@SiO_2 modified metallic Cu	-0.67	2.13	47.05	N.A.	4.57	7.69	13.20	N.A.
	-0.72	2.2	51.29	N.A.	4.01	5.45	19.75	N.A.
	-0.80	0.58	47.57	0.78	5.85	5.02	18.45	N.A.
	-0.92	0.75	42.50	5.21	10.21	4.11	15.78	N.A.
	-1.03	0.43	30.20	16.17	17.48	2.01	12.37	N.A.

Table S5. Faradaic efficiencies of CO_2RR products at various applied voltage for AuNPs@SiO_2 modified metallic Cu.

Sample	Voltage (vs RHE)	FE_{CO} (%)	FE_{C2H4} (%)	FE_{CH4} (%)	FE_{H2} (%)	FE_{HCOOH} (%)	FE_{C2H5OH} (%)	FE_{C3H7OH} (%)
AuNPs@SiO_2 modified metallic Cu	-0.67	2.64	47.35	N.A.	7.02	5.91	14.06	N.A.
	-0.72	1.99	51.87	N.A.	6.06	5.33	18.39	N.A.
	-0.80	1.43	50.52	N.A.	8.18	4.18	16.57	N.A.
	-0.92	0.47	40.80	2.58	12.37	4.01	13.00	N.A.
	-1.03	0.63	31.73	16.01	18.42	1.97	9.38	N.A.

References

(1) Ye, X.; Gao, Y.; Chen, J.; Reifsnyder, D. C.; Zheng, C.; Murray, C. B. Seeded growth of monodisperse gold nanorods using bromide-free surfactant mixtures. *Nano Lett.* **2013**, *13* (5), 2163-2171. DOI: 10.1021/nl400653s.

(2) Li, J. F.; Tian, X. D.; Li, S. B.; Anema, J. R.; Yang, Z. L.; Ding, Y.; Wu, Y. F.; Zeng, Y. M.; Chen, Q. Z.; Ren, B.; et al. Surface analysis using shell-isolated nanoparticle-enhanced Raman spectroscopy. *Nature Protocols* **2013**, *8* (1), 52-65. DOI: 10.1038/nprot.2012.141.
